# Total Extirpation of the Prostate

**Published:** 1901-09-21

**Authors:** 


### TOTAL EXTIRPATION OF THE PROSTATE.

THE lawyers are apt to say that the essence of a crime lies in the intent rather than in the act committed, and it is possible that if the question now being discussed with a certain amount of acrimony as to priority of invention of Mr. Freyer's "new operation" for the extirpation of the prostate were considered in the same way, much of the difference would vanish. As we described in a recent article, Mr. Freyer would treat certain cases of enlarged prostate by enucleation of the gland. He maintains that the prostate is from the first a double gland, and that the obstruction to the outflow of urine is due to the urethra being compressed between its two halves which, although they grow together, are in origin distinct. Having reached that point he advises that an operation should be done, the object and intent of which is the enucleation of these two halves, knowing that if that be done the urethra will be set at liberty. With the urethra itself he endeavours not to interfere. This is his "new operation." No sooner, however, did Mr. Freyer claim that this operation was "new" than he was assailed from many quarters. It was pointed out, with great justice, that the prostate had been enucleated many times before, and with striking success. Although the late Mr. McGill in his earlier cases attacked the obstructing portion of the prostate, it is clear that later both he and those who followed him went much further and enucleated considerable portions of the gland, perhaps the whole of it. Hence they are probably right in maintaining that what Mr. Freyer does they had done, and that in that sense the mere enucleation of the prostate is not a new thing. Moreover, it seems evident that Mr. Freyer much under estimated the success which some operators had met with in their operative proceedings. A man must be very sure of his ground before he sets up a claim for novelty nowadays. Still novelty may lie in the intent as well as in the thing done, and from a careful perusal of all the letters which have been published on the subject, while we cannot but regret the tone in which the discussion has been carried on, we come to the conclusion that if we take a lesson from the lawyers and consider the intent of the operation instead of concentrating our attention upon the actual details of the proceedings, Mr. Freyer is right in speaking of it as a "new operation." The others aim at removing the obstruction, and, although they often remove a good deal more, if they have removed the obstruction they have done what they set out to do. Mr. Freyer on the other hand, putting on one side the question what particular part of the prostate may at that particular time be doing the mischief, seems to say, "If I can but extirpate this prostate the man will be cured"; so his aim is to extirpate the prostate, and we cannot but feel that looking at the matter in this light his operation is new.

